# *Vorticella*: A Protozoan for Bio-Inspired Engineering

**DOI:** 10.3390/mi8010004

**Published:** 2016-12-26

**Authors:** Sangjin Ryu, Rachel E. Pepper, Moeto Nagai, Danielle C. France

**Affiliations:** 1 Department of Mechanical and Materials Engineering, University of Nebraska-Lincoln, Lincoln, NE 68588, USA; 2 Department of Physics, University of Puget Sound, Tacoma, WA 98416, USA; rpepper@pugetsound.edu; 3 Department of Mechanical Engineering, Toyohashi University of Technology, Toyohashi 441-8580, Japan; nagai@me.tut.ac.jp; 4 National Institute of Standards and Technology, Boulder, CO 80305, USA; danielle.france@nist.gov

**Keywords:** *Vorticella*, oral cilia, feeding current, contractile stalk, spasmoneme, Ca^2+^-powered contraction, bioinspired engineering

## Abstract

In this review, we introduce *Vorticella* as a model biological micromachine for microscale engineering systems. *Vorticella* has two motile organelles: the oral cilia of the zooid and the contractile spasmoneme in the stalk. The oral cilia beat periodically, generating a water flow that translates food particles toward the animal at speeds in the order of 0.1–1 mm/s. The ciliary flow of *Vorticella* has been characterized by experimental measurement and theoretical modeling, and tested for flow control and mixing in microfluidic systems. The spasmoneme contracts in a few milliseconds, coiling the stalk and moving the zooid at 15–90 mm/s. Because the spasmoneme generates tension in the order of 10–100 nN, powered by calcium ion binding, it serves as a model system for biomimetic actuators in microscale engineering systems. The spasmonemal contraction of *Vorticella* has been characterized by experimental measurement of its dynamics and energetics, and both live and extracted *Vorticellae* have been tested for moving microscale objects. We describe past work to elucidate the contraction mechanism of the spasmoneme, recognizing that past and continuing efforts will increase the possibilities of using the spasmoneme as a microscale actuator as well as leading towards bioinspired actuators mimicking the spasmoneme.

## 1. Introduction

### 1.1. Overview of Vorticella

Among various microorganisms, protozoa have been widely studied as model systems for biology, physics, engineering and biomimetics. They form a diverse group of unicellular eukaryotic organisms including ciliates, flagellates and amoeboids. Among such protists, *Vorticella* has drawn the attention of scientists since Anthony van Leeuwenhoek first described its unique motility in his letter dated 9 October 1676 [1,2]. In a recent study, *Vorticella* was discovered in a fossil that is more than 200 million year old, bolstering the fossil record of soft-bodied organisms [3]. While we primarily describe biomechanical and biomimetic aspects of *Vorticella*, uncovered since the 1950s, it is worth noting that *Vorticella* has played a long and ongoing role in many fields of biological, physical and engineering study. 

**Figure 1:**
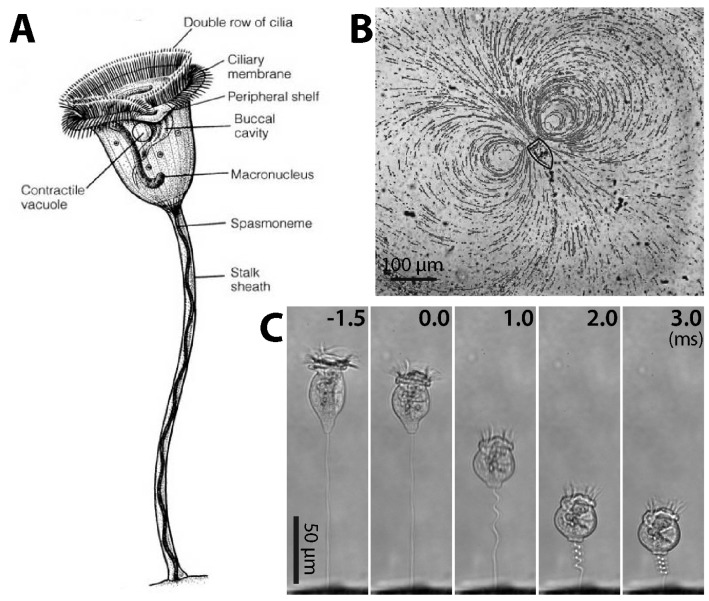
Sessile ciliate protozoan *Vorticella*. (**A**) Structure of *Vorticella convallaria* (reproduced from [7]); (**B**) pathlines of water flow generated by cilia beating of *V. convallaria* (reproduced from [8]); (**C**) sequential images of stalk contraction of *V. convallaria* (reproduced from [9]). The stalk begins to coil at 0 ms.

*Vorticella* is a suspension-feeding ciliate that lives in two forms: free swimming telotroch and sessile stalked trophont [4]. A sessile *Vorticella* consists of the zooid (inverted-bell-shaped cell body; usually about 30–40 μm in diameter when contracted) and the stalk (3–4 μm in diameter and about 100 μm in length) (Figure 1A). The zooid has two bands of cilia around the peristome, the mouth-like part of the zooid, which are used for suspension feeding. The cilia of the inner band generate water flow to draw food particles toward the zooid (Figure 1B), and these particles are filtered by the cilia of the outer band. An example of such a feeding current observed in the laboratory shows a vortex (Figure 1B), and it was observed that this flow could move micro-diameter particles at least 450 μm away from the peristome with the maximum flow velocity of 360 μm/s [5,6]. 

In the sessile form, the vertex or scopula of the zooid is connected to the proximal end of the stalk while the distal end is rooted on a habitat surface (Figure 1A). Therefore, the stalk tethers the zooid to the habitat. The stalk also coils very quickly and moves the zooid toward the surface through the action of its contractile organelle, the spasmoneme (Figure 1C). Afterwards, the stalk slowly relaxes and moves the zooid away from the surface over a few seconds [10,11,12]. Although the stalk contraction-relaxation cycle of *Vorticella* has been hypothesized as a way either to avoid dangers, or to mix the surrounding fluid, no explanation is completely satisfactory. 

During stalk contraction, *Vorticella* stops ciliary beating and contracts the zooid to a nearly spherical shape, and the stalk shortens to 20%–40% of its extended length in less than 10 ms (Figure 1C). Therefore, *Vorticella* contracts at the average rate of 10–20 mm/s, and its zooid reaches a maximum speed of 60–90 mm/s [9,12,13,14]. In terms of specific velocity (body length/s), this maximum speed corresponds to ~1200 body length/s, making *Vorticella* among the fastest living creatures using this metric [12]. It has also been estimated that in terms of specific power (power dissipation per mass) the stalk of *Vorticella* performs better than both automotive engines and striated muscle [15]. Recent measurements showed that live spasmonemes could generate a peak contractile force of ~30 nN with maximum power of ~1.6 nW during normal contraction, and isometric tension of 150–350 nN [9,16]. 

In addition to its remarkable motility, the energy source for the stalk contraction makes *Vorticella* unique. While most biological contractile systems depend on ATP (adenosine triphosphate) for their biochemical fuel, the *Vorticella* spasmoneme is powered by calcium ions [17]. In the absence of ATP, extracted *Vorticella* stalks can repeat the contraction–relaxation cycle and generate contractile force, driven only by external calcium ions diffusing into the spasmoneme [17,18,19,20,21,22,23]. Because of its surpassing contractility and unique energy source, therefore, the *Vorticella* stalk is regarded as a candidate for Ca^2+^-powered cell motility and biomimetic actuating materials [24,25,26,27,28,29]. 

### 1.2. Similar Microorganisms

**Figure 2:**
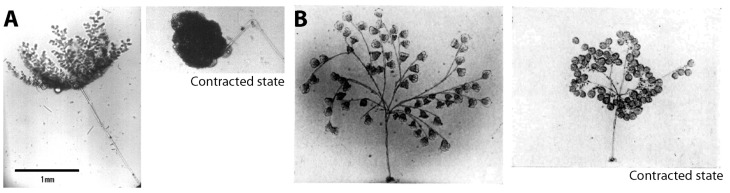
Other protozoa with Ca^2+^-driven stalk contraction. (**A**) *Zoothamnium* (reproduced from [58]); (**B**) *Carchesium* (reproduced from [59]).

There are various other microorganisms which contract using Ca^2+^, such as *Stentor* and *Paramecium*. Among them, *Zoothamnium* and *Carchesium* are close to *Vorticella* in that these species also have contractile stalks (Figure 2). These larger ciliates have one main stalk with many branches ending in zooids, which is a major difference from *Vorticella*, which has only a single stalk and zooid. Upon stimulation, *Zoothamnium* contracts the entire colony into one large globule and then folds the main stalk (Figure 2A). In contrast, *Carchesium* contracts each zooid and its stalk separately (Figure 2B). This difference is due to a structural difference of the spasmoneme between the two species: the *Zoothamnium* spasmoneme is continuous among zooids in the colony, whereas the *Carchesium* spasmoneme is discontinuous between zooids. Because *Zoothamnium* and *Carchesium* have bigger spasmonemes than *Vorticella* does (the spasmoneme of *Zoothamnium* is about 30 μm in diameter and 1 mm long [30]), they have also been employed for studying the spasmonemal contraction [10,30,31,32,33,34,35,36,37,38,39,40,41,42,43,44,45,46,47,48,49,50,51,52,53,54,55,56,57]. 

### 1.3. Focus of this Review

In this review, we focus on *Vorticella* in the sessile form and summarize previous studies about its beating cilia and contractile stalk. Because the most recent reviews on *Vorticella* focus on its biological aspects and stalk contraction [4,15,60], our review is complementary to them, providing an in-depth review of *Vorticella* from cellular mechanics and bio-inspired engineering perspectives. Furthermore, we intend to introduce *Vorticella* as a promising biological model system for bio-inspired engineering and biomimetics. 

This review consists of two main parts. In the first part, we introduce the common features of cilia and review previous studies on the flow-generating-capability of the *Vorticella* cilia. Then, we discuss possibilities of using the *Vorticella* cilia for engineering applications. In the second section, we focus on the stalk of *Vorticella* and its Ca^2+^-powered contraction, including relevant studies on *Zoothamnium* and *Carchesium*. We also discuss how *Vorticella* with the contractile stalk can be used in microscale systems as an actuator and as a model for bio-inspired engineering.

## 2. Cilia and Cilia-Generated Flow of *Vorticella*

### 2.1. Structure of Cilia Rows

**Figure 3:**
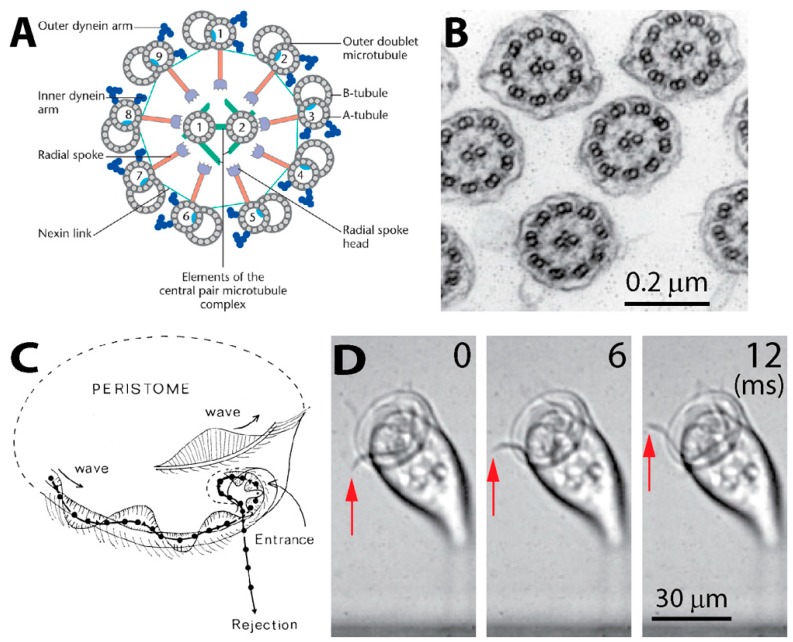
Oral cilia of *Vorticella*. (**A**) Cross-sectional view of the common structure of the cilium (reproduced from [63]). A cilium consists of nine circumferential doublet microtubules and one central pair of single microtubules (9 + 2 pattern). Dynein arms generate sliding motions between doublet microtubules, which bends the cilium; (**B**) cross-sectional view of the cilia of *Vorticella*. (reproduced from [67,68]); (**C**) a metachronal wave of cilia around the oral part of *Vorticella* (reproduced from [5]). The motion of the black particle shows transport of particles near the peristome; (**D**) red arrows show the metachronal ciliary wave of *V. convallaria*.

In his first description of *Vorticella*, Leeuwenhoek thought that *Vorticella* had two horns moving like horse ears near the oral part [1,2]. What he actually observed were beating oral cilia generating water flow. These cilia have a structure common to eukaryotic cells: each cilium includes a bundle of microtubules extending continuously for the length of the cilium with the so called “9 + 2” pattern (Figure 3A,B) [61,62]. As the stiffest cellular filament, a microtubule is a hollow tube-like polymer of a globular protein tubulin. In this 9 + 2 arrangement, a pair of singlet microtubules is surrounded by nine doublet microtubules. 

Ciliary beating is characterized by a series of bends of the cilium, which depends on the sliding of adjacent doublet microtubules caused by dynein, one of the microtubule motor proteins [63]. Arms of dynein molecules are attached periodically along the length of the microtubule, and these arms of one doublet walk along the adjacent doublet powered by hydrolysis of ATP [62]. While each cilium beats periodically, their synchronized motion forms metachronal waves along the periphery of the oral part (Figure 3C,D), which causes a circulating current in the surrounding water (Figure 1B) [5]. This ciliary metachronal wave is a common motility mechanism among microorganisms [64,65,66]. 

### 2.2. Ciliary Performance

Using *Vorticella* as a model protozoan for microscale fluid control requires evaluating its ciliary performance and understanding the flow caused by the cilia. Such studies experimentally measured the field and strength of the ciliary flow of *Vorticella* by tracing particles in the flow. Fluid dynamics modeling has also been employed to model the ciliary flow based on the fact that the flow is dominated by viscosity. Characterizations made with both experiments and theoretical models are discussed here. 

#### 2.2.1. Flow Measurement 

Sleigh and Barlow first examined the feeding current of *V. convallaria* by tracing micron-sized particles in the vicinity of the zooid using high-speed cinematography [5]. They observed that 17 μm-long cilia beat at about 50 Hz with the tip speed of 4 mm/s while particles moved at 2.5 mm/s near ciliary tips. This ciliary beating enabled *V. convallaria* to draw particles 450 μm away from the oral part and to collect particles in a volume of about 0.3 mm^3^. 

Later, Vopel et al. measured the velocity of the feeding current of a marine *Vorticella* using a flow microsensor [69]. The measured flow velocity was about 18 mm/s and 2.6 mm/s at a horizontal distance of 50 μm and 350 μm from the oral part, respectively, and 0.8 mm/s at the stalk base. Having observed that the *Vorticella* moved the surrounding seawater at least 400 μm from the zooid, which agrees with Sleigh and Barlow’s observation, Vopel et al. suggested that the ciliate could generate sufficient feeding flow by raising the zooid above the substrate by the stalk. 

**Figure 4:**
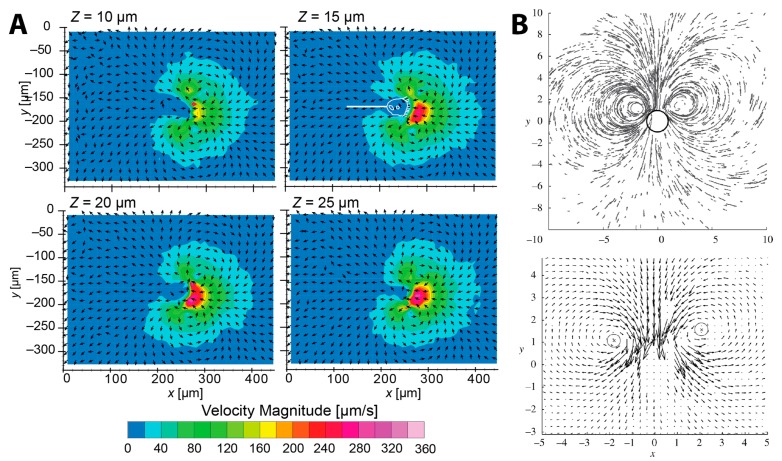
Feeding current of *Vorticella*. (**A**) μ-PIV measurements of the feeding current of *V. picta* (reproduced from [6]). 2D flow fields of *x*- and *y*-velocity components were measured at various *z* planes; (**B**) particle paths and flow field obtained from the feeding current of *V. convallaria* shown in Figure 1B. The circle in the top figure represents the zooid, and the two circles in the bottom figure show the center of eddies (reproduced from [8]).

Recently, Nagai et al. rigorously measured the two-dimensional (2D) velocity components of the feeding current of *V. picta* using the confocal micro-particle image velocimetry (μ-PIV) [6]. Their reconstructed flow velocity field showed the same double-vortex structure (Figure 4A) seen by Sleigh and Barlow [5]. Although Sleigh and Barlow extrapolated an axisymmetric toroidal vortex structure from these twin vortices, truly three-dimensional flow measurement is still required to validate the seemingly toroidal feeding current of *Vorticella*. 

Nagai et al. observed the maximum flow speed of about 360 μm/s occurring in front of the peristome, and evaluated the volume flow rate toward the oral part to be about 3 × 10^−4^ mm^3^/s. This maximum flow speed is about one order of magnitude lower than that of the previous two studies. This is because flow speed measurements depend on the region of the measurement in the vicinity of cilia and the resolution of imaging. It is noticeable that 1/3 of this inflow was carried from fresh water whereas 2/3 was recirculated. Therefore, *V. picta* appeared to clear water at a rate of 0.36 mm^3^/h and to collect particles from a fluid volume of 1.7 × 10^−3^ mm^3^.

Pepper et al. also measured the feeding current of *V. convallaria* using particle tracking velocimetry (PTV) (Figure 1B and Figure 4B) [8]. Individual *V. covallaria* cells were anchored to the thin edge of a coverslip, confined between two closely-spaced surfaces. Having observed that increasing the spacing between the confining surfaces increased the size of the observed vortices, Pepper et al. suggested that the feeding flow in nature without confining boundaries nearby might differ significantly from the dual-vortex structure seen for *Vorticella* sandwiched between surfaces. A full 3D measurement of the feeding current around a *Vorticella* without nearby boundaries has not yet been made. 

#### 2.2.2. Fluid Dynamic Model

Since *Vorticellae* are small and their feeding current is relatively slow, they feed in the regime of low Reynolds number flow (i.e., Stokes flow) [70]. The Reynolds number is the ratio of inertial to viscous forces in a fluid flow, and is defined as *Re* = *UL*/*ν*. Here, *U* and *L* are a characteristic velocity and length of the flow, and *ν* is the kinematic viscosity of the fluid. For *Vorticella* in water (zooid diameter *L* ≈ 40 μm and feeding flow speed *U* ≈ 100 μm/s), a typical Reynolds number of the feeding current is 4 × 10^−3^. Thus, viscous forces dominate over inertia forces in the feeding current of *Vorticella*. 

**Figure 5:**
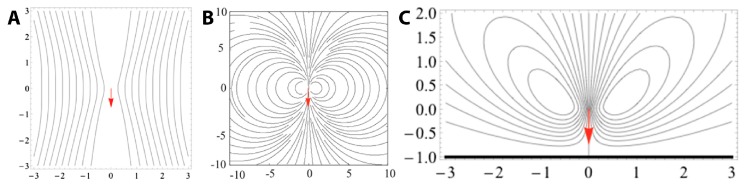
Streamlines around a stokeslet in various geometries. The red arrow represents the direction of the stokeslet force and thus the orientation of the *Vorticella* zooid axis (arrow points from the peristome to the scopula). (**A**) Stokeslet in free space; (**B**) stokeslet between two parallel planes (reproduced from [8]); (**C**) stokeslet pointing perpendicular to a single plane boundary, which is indicated by the bold black line.

The feeding current of microscopic suspension feeders including *Vorticella* is often modeled as a single stokeslet, a point force in Stokes flow [8,71,72,73,74,75,76,77,78,79,80,81,82]. A stokeslet in free space (Figure 5A) does not have the characteristic toroidal flow pattern seen in Figure 4, whereas several different models of *Vorticella* feeding between two closely-spaced parallel boundaries result in such recirculating flow with tight, circular streamlines (Figure 5B) [8,74,75]. This theoretical observation suggests that the circular vortices are due to the two boundaries sandwiching the organism, which are glass surfaces present in most experimental observations of *Vorticella* feeding flow [8,71,74]. Indeed, the feeding flow field produced by *Vorticella* is likely to be determined mostly by the geometry of nearby boundaries [74].

A more realistic model for feeding *Vorticellae* is likely a stokeslet forcing fluid towards a single plane which represents the habitat substrate. Having modeled *Vorticella* as such a stokeslet, Blake and Otto found that the feeding current consists of a toroidal vortex around the organism (Figure 5C), but not with the characteristic circular shape found in experiments and with a stokeslet between two boundaries [72].

Recently, Pepper et al. showed that the single stokeslet model with nearby boundaries agrees well with experimentally-measured feeding flow fields around *Vorticella* [8]. They also confirmed that the size of these eddies is determined by the distance between the two boundaries, which indicates that the eddies would not be present if no boundaries were nearby, as shown by Figure 5A. Moreover, they estimated that the total force exerted by the cilia of *V. convallaria* on the surrounding water is 0.05–0.5 nN [80,83,84]. It has also been shown that when only one boundary is nearby, the fluid flow changes dramatically depending on the angle of *Vorticella* to the surface [84]. Therefore, effects of nearby boundaries need to be considered when utilizing the ciliary flow of *Vorticella* and other microorganisms.

### 2.3 Engineering Applications

It has been suggested that the biological motors of microorganisms can be used or mimicked in microsystems to reduce the overall size of devices, and cilia beating has been envisaged as a potential strategy for inducing fluid flow and mixing in microsystems [85]. Cilia-mimicking devices such as artificial cilia have succeeded in generating pumping and mixing [86,87,88,89,90,91,92,93,94,95,96], but they require external power sources for operation, which enlarges the system. In contrast, cellular cilia do not require such external sources because they convert in situ biochemical energy to mechanical work [97], which seems ideal for components of microsystems. For instance, live carpets of flagellated bacteria have been used to produce directional flows and thus to transport fluid [98,99,100]. 

**Figure 6:**
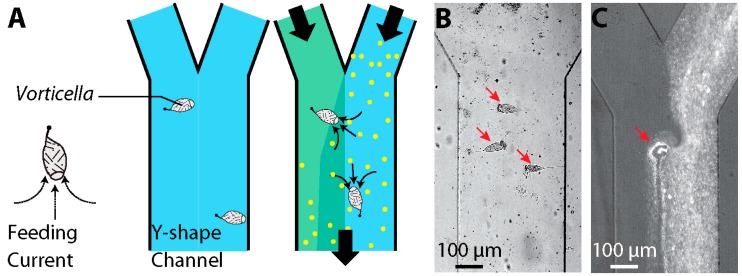
*Vorticella* as a microfluidic mixer (reproduced from [101]). (**A**) Schematic of using the *Vorticella* cilia for micromixing; (**B**) *V. convallaria* cells in a Y-channel; (**C**) transport of microparticles by a single *V. convallaria* cell, and resultant mixing between two streams in the Y-channel.

Similarly, the cilia of live *Vorticella* can function as a driving source for fluid motion in microfluidic devices. Recently, Nagai et al. used the oral cilia motion of live *V. convallaria* in a microchannel for mixing enhancement (Figure 6) [101,102]. Several *V. convallaria* cells on a channel surface caused active mixing between two streams in a Y-channel based on fluid transport generated by their cilia. Another possible application could be fluid pumping, for which multiple *Vorticellae* generate directional flow and thus transport target objects in the flow direction. For better performance, *Vorticellae* can be patterned in microsystems using a guiding flow into the system [101,103]. However, drawbacks exist in using live *Vorticellae* in the microsystem, such as providing nutrients and removing waste for sustainable culture of the microorganisms, controlling their location, and packaging fluidic systems for controlling microenvironments.

## 3. Stalk and Ultrafast Contraction of *Vorticella*

### 3.1. Structure and Coiling of the Stalk

Arguably, the vast majority of the attention paid to sessile *Vorticella* has focused on the contractility of its stalk and thus the stalk structure. Although the coiling stalk of *Vorticella* was witnessed centuries ago under the first microscope [1,2], it has taken more modern investigations at various length scales to reveal the structural elements that enact *Vorticella*’s rapid movement. 

**Figure 7:**
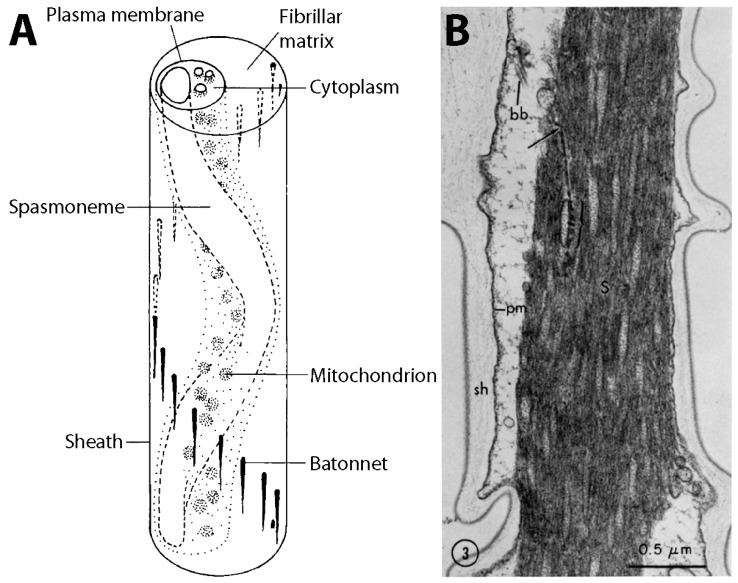
Structure of the *Vorticella* stalk. (**A**) Schematic of the stalk (reproduced from [37]); (**B**) cross-sectional micrograph of the stalk showing that the spasmoneme is composed of 3–4 nm-diameter fibrils (reproduced from [67]).

The *Vorticella* stalk consists of a fibrous contractile organelle (the spasmoneme), a relatively robust sheath, a matrix of tiny fibers (the fibrillar matrix), and rod-like bundles of filaments (the bâtonnets) stiffening the sheath (Figure 7A). The spasmoneme runs through the entire length of the stalk as a left-handed helix [21], whereas the bâtonnets run as a right-handed helix, always located on the opposite side of the sheath from the spasmoneme. The spasmoneme is intracellular, connected with the zooid whereas the sheath material is extracellular and primarily considered structural in nature. 

Coiling of the stalk involves the mechanical interplay of these elements. As the stalk coils, the spasmoneme takes a shorter path through the coil while the bâtonnets remain on the outside of the coil indicating the least deformable part of the sheath and stalk [34,37]. During the course of rapid contraction, the spasmoneme twists itself, and the stored strain is relaxed by the rotation of the zooid at the end of contraction [12]. Re-extension or relaxation of the stalk is also a mechanical interplay of these elements. The roles of each element have not been precisely defined, although the elastic restoring force of the deformed sheath was suggested to be responsible for the stalk re-extension [13]. 

The fibrous structure of the spasmoneme was first shown in transmission electron microscope (TEM) images in the early 1970s (Figure 7B) [37,104]. These early observations revealed hundreds of filaments, each roughly 2–4 nm in diameter, in the contracted spasmoneme. These fibrils are interspersed with tubules which are roughly 50 nm in diameter with ~250 nm spacing. These round membranated structures have been proposed as mitochondria and/or calcium storage sites [37,104].

**Figure 8:**
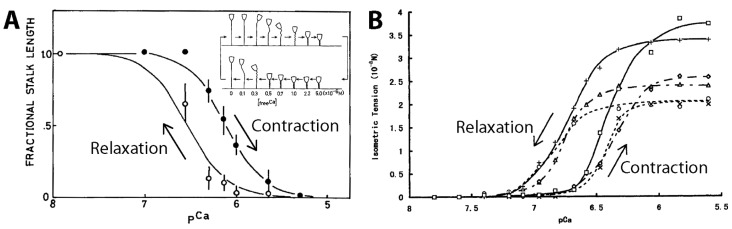
Calcium-dependency of *Vorticella*’s stalk contraction. (**A**) The contraction-relaxation cycle of the extracted stalks of *V. convallaria* responding to changes in [Ca^2+^]_free_ (reproduced from [20]). Inset: Shape changes of *V. convallaria* depending on [Ca^2+^]_free_ (reproduced from [58]); (**B**) isometric tension developed by the extracted stalk of *V. convallaria* responding to changes in [Ca^2+^]_free_ (reproduced from [23]). Here, $\mathrm{pCa}\;=-\log_{10}{\left[{\mathrm{Ca}}^{2+}\right]}_{\mathrm{free}}$.

As previously mentioned, calcium ions induce and power the spasmonemal contraction [17]. In the absence of ATP, the permeabilized stalk can coil if the free Ca^2+^ concentration of the medium ([Ca^2+^]_free_) is higher than 10^−6^ M (Figure 8A) [19,20,23]. This is because external calcium ions can diffuse into the stalk and trigger the spasmoneme. The permeabilized stalk remains extended when [Ca^2+^]_free_ < 10^−8^ M. The stalk can repeat the contraction-relaxation cycle and generate tension upon changes in [Ca^2+^]_free_, although it loses contractility over repeated cycles (Figure 8B). 

Should the membranous tubules seen by TEM be proven to contain high concentrations of calcium, they could feasibly serve as energy reservoirs for the spasmoneme [4,37]. As a contraction is triggered, calcium is thought to be released from the tubules [37,105]. Relaxation of the stalk then requires that calcium be pumped back to its storage organelles by ATP-dependent pumps [48,56]. These two processes occur on vastly different time scales: milliseconds for contraction and seconds for relaxation. 

### 3.2. Contraction Mechanism

**Figure 9:**
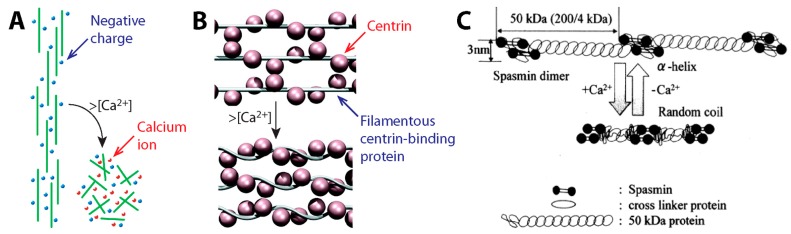
Suggested models for the *Vorticella* spasmoneme. (**A**) Electrostatic model based on the concept of the entropic spring (reproduced from [15]); (**B**) two-component model suggested for the centrin-based infraciliary lattice of *Paramecium* (reproduced from [107]); (**C**) two-component model considering the protein components (spasmin and spaconnectin) of the spasmoneme (reproduced from [58]).

When considered together, the stalk elements point towards *Vorticella* stalk coiling being carried out by a fibrous, ordered substructure of the spasmoneme actuated by calcium ions. Two prominent models were proposed that relied heavily on the filamentous structure of the spasmoneme. In the electrostatic model (Figure 9A) [17], the nanofibrils of the spasmoneme are negatively charged, so they are aligned in parallel due to electrostatic repulsive force among the filaments. Binding of calcium ions neutralizes the charged filaments, which results in the collapse of the filaments and thus contraction of the spasmoneme. However, the electrostatic model does not consider the Ca^2+^-specificity of the spasmoneme [46,48]. The spasmonemal contraction can be induced by a divalent cation of around the same size as Ca^2+^ whereas electrostatic screening by monovalent salt ions cannot do so [106]. The second model assumes the spasmoneme to be a rubber-like material [38,44,56], wherein calcium binding induces the intramolecular folding of contractile elements or peptides. A common drawback of the above models is that they do not consider the biochemistry of the spasmoneme components.

Routledge et al. first identified the major protein component of the *Vorticella* spasmoneme, a 20 kilodalton (kDa) Ca^2+^-binding protein termed spasmin [40]. Spasmin is negatively charged and becomes more hydrophobic upon calcium binding [40,48,49,108]. Because of its abundance in the spasmoneme and affinity for calcium, spasmin is widely considered to be the actuating protein of the spasmoneme. Spasmin appears to have a binding partner in the spasmoneme, a 50 kDa spasmin-receptor protein named spaconnectin [55,56,109,110]. This protein pair is also found in the spasmoneme of *Carchesium* and *Zoothamnium* [110]. However, the roles of spasmin and spaconnectin in *Vorticella* contractility have yet to be identified.

Centrin, a homolog of spasmin, provides clues to elucidate the contraction mechanism of the spasmoneme. As a eukaryotic signature protein, centrin is a highly conserved Ca^2+^-binding protein found contractile organelles of some eukaryotic cells [55,111,112,113,114]. Because centrin does not readily form nanofibers on its own, its binding partners have been identified which have multiple repeated conserved sites for centrin binding and higher fibril-forming potential [115,116,117,118]. For instance, the contractile infraciliary lattice (ICL) of *Paramecium* contains centrin and a centrin-binding protein named PtCenBP1 (Figure 9B) [107]. It has been suggested that the Ca^2+^-responding nanofilaments of the ICL consist of PtCenBP1 molecules containing repeated centrin-binding motifs, so multiple centrin molecules bind to each extended PtCenBP1 molecule at low [Ca^2+^]_free_. Upon influx of Ca^2+^, centrin molecules undergo conformational changes, which causes shortening of the filaments and thus the contraction of the ICL [107]. 

Recently, a similar two-component model has been proposed for the spasmoneme based on spasmin and spaconnectin (Figure 9C) [58]. This model assumes that the spasmonemal nanofibrils consist of an *α*-helix of spaconnectin joining a pair of spasmin molecules. Upon increase in [Ca^2+^]_free_, spaconnectin molecules become random coils, so the filaments shorten. This two-component model leaves room to accommodate several key aspects from the experimental evidence: specificity for calcium and hydrophobic interactions that become available when spasmin binds calcium. Identifying the contraction mechanism of the spasmoneme based on the roles of its protein components is crucial for exploiting the Ca^2+^-induced contraction of the spasmoneme in bio-inspired engineering.

### 3.3. Contractile Performance 

Studies on the stalk contractility of *Vorticella* not only give us insights into how the spasmoneme operates, but also frame the motor capabilities of the spasmoneme as a model for bioinspired actuators [26]. Such studies measured key parameters including contraction speed, coiling propagation speed, contractile force (or tension), mechanical work, and power, using both live and extracted *Vorticella* cells. High-speed imaging techniques have enabled observations of the millisecond contraction sequence of live *Vorticella*. In contrast, extracted cells have enabled easier manipulations and observations of the spasmonemal contraction as shown in Figure 8, and if stored frozen in glycerin, remain contractile for up to several months [19]. Observations made with both live and extracted cells are discussed here. 

#### 3.3.1. Dynamics of Stalk Contraction

Two types of speed measurements have been taken for *Vorticella*: the contracting speed of the whole stalk, which equals the moving speed of the zooid during stalk contraction, and the propagation speed of the contraction onset, which proceeds from the zooid to the distal end of the stalk (Figure 1C). The former is defined by the capabilities of the contractile machinery as a whole, while the latter is believed to be dictated by the molecular signaling mechanism that drives calcium release within the spasmoneme, thereby instigating contraction. 

Various imaging methods have been employed for time-resolved investigations of live *Vorticella*’s contraction. To the best knowledge of the authors, Ueda made the first measurements of the contraction speed of *Carchesium polypinum*, which was 17.8–24.7 cm/s, using a photographic method for which a detailed description was not given [32]. Later, Sugi projected the image of the contracting stalk of *C. polypinum*, with carbon granules attached, through a narrow slit onto a rotating photographic paper roll, and measured that the contraction propagation speed was 20–50 cm/s, which is higher than the contraction speed [33]. A similar approach was employed for *Vorticella* by Katoh and Naitoh, and the measured contraction speed (6.7 cm/s) was lower than that of *C. polypinum* [11]. These measurements are summarized in Table 1 along with the latent period (the time between observed cell body contraction and the onset of stalk contraction). 

**Figure 10:**
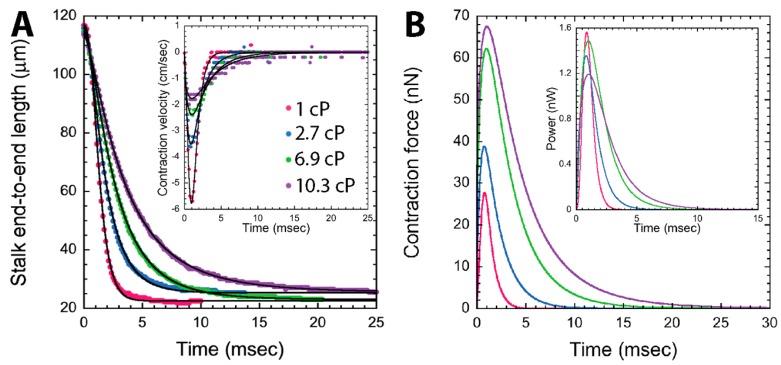
The stalk contraction dynamics and energetics of *V. convallaria* (reproduced from [9]). (**A**) Time courses of the stalk length and contraction speed (inset); (**B**) contractile force and power (inset) calculated from the velocity profile. 1 cP (centipoise) is the dynamic viscosity of water (= 0.1 Pa·s). As the medium viscosity increased, *V. convallaria* contracted slower with higher contractile force while the peak power was limited.

Early observations with high-speed cameras were instigated by Jones et al. with *V. difficilis*, *V. campanula*, and *Carchesium* [119]. Later, the dynamics of *V. convallaria*’s stalk contraction was extensively investigated by Moriyama et al. [12], Upadhyaya et al. [14], Ryu and Matsudaira [9] and Kamiguri et al. [120]. In these video microscopy studies, a *Vorticella* lying in the focal plane of the microscope was imaged (Figure 1C), and the time course of contraction speed was obtained from the stalk length change directly measured from sequential images captured during contraction (Figure 10A). Also, the propagation speed of stalk coiling could be measured by measuring a time difference in coiling initiation between two points on the stalk. As summarized in Table 1, *Vorticella* in water reaches the maximum contraction speed of 1.5–9 cm/s at 1–2 ms after the initiation of the stalk contraction, and its stalk coiling propagates at a speed of about 10 cm/s and is completed in 4–9 ms. 

#### 3.3.2. Model-Based Tension Measurements 

Given a measure of contraction speed, the contractile force of the spasmoneme can be estimated using fluid dynamics modeling. In the model, the contracted zooid is assumed to be a solid spherical body moving in a viscous liquid. During translational motion caused by the coiling stalk, the zooid experiences the tension from the stalk and the drag force from the surrounding liquid. Although the net force of the two components accelerates the zooid, the contractile force can be equated to the drag because the resultant inertial force of the zooid is negligible due to the small mass of the zooid. Therefore, the spasmonemal tension can be determined from fluid dynamics calculations. 

Amos first utilized this modeling approach for *Vorticella* using Stokes’ law [13]. This drag formula calculates drag force on a sphere moving at low Reynolds numbers (*Re* = 2*RU*/*ν* << 1) to be 6*πμRU*, where *μ* is the liquid viscosity, *R* and *U* are the radius and speed of the zooid, respectively. Using Jones et al.’s study [119], he estimated that the mean contraction force and minimum work of the *Vorticella* spasmoneme were 8.7 nN and 0.69 pJ, respectively [13,44]. Moriyama et al. [12] and Upadhyaya et al. [14] employed the same approach and high-speed imaging, and they estimated the peak contraction force of *V. convallaria* to be 55.8 nN and ~30 nN, respectively (Table 2). 

Later, computational fluid dynamics (CFD) simulations were utilized by Ryu and Matsudaira for more rigorous estimations. They modeled a contracting *V. convallaria* as a sphere moving toward a solid plane, simulated water flow caused by the sphere translating with the measured profile of the stalk length change, and then calculated the drag on the zooid from the simulated water flow. Their simulation showed that *V. convallaria* contracted in water with the maximum tension of 28 nN and maximum power of 1.6 nW, and that its spasmoneme converted chemical energy from Ca^2+^-binding to mechanical work of 1.64 pJ with energy conversion efficiency of 8% (Figure 10B). Here, the efficiency is a ratio of mechanical work done by the spasmoneme to chemical energy available from calcium binding.

#### 3.3.3. Experimental Tension Measurements 

Hydrodynamic force estimations suggest the lower limit of the contractile force of the *Vorticella* spasmoneme because more force must have been generated to deform the sheath and to overcome the internal resistance of the stalk [48]. In contrast, the upper limit on contractile force is the isometric tension of the spasmoneme that is generated when the stalk is not allowed to shorten. Therefore, measuring the upper limit involves applying external resistances to *Vorticella* to hinder full contraction. 

Both the high contraction speed and small size of *Vorticella* make the manipulations needed for isometric tension measurements challenging. Size difficulties were circumvented by using *Carchesium* and *Zoothamnium*, and the tensile stress of their spasmonemes was measured to be 6–78 kPa (1 Pa = 1 N/m^2^) [31,38,39,42]. Speed difficulties were overcome by using extracted stalks. Moriyama et al. induced tension of the extracted stalk of *V. convallaria* by increasing [Ca^2+^]_free_ while fixing both ends of the stalk, which measured isometric tension (Figure 8B) [23]. The measured isometric tension was 40 nN on average and 120 nN at maximum, which corresponds to tensile stresses of 35–51 kPa.

One method attempted to directly measure the isometric tension of live *Vorticella* was to retard stalk contraction by increasing the medium viscosity [9,14]. Under increased viscous drag, *V. convallaria* took a longer time to complete stalk contraction with lower speed, and generated higher contractile force to overcome increased resistance (Figure 10). Upadhyaya et al. first noticed that power dissipation of multiple *V. convallaria* cells, which is the product of force and velocity, was 1–3 nW even with significant increase in the medium viscosity [14]. Recently, Kamiguri et al. used various polymer additives and made similar observations [121]. The idea of power-limited contraction was more rigorously examined by Ryu and Matsudaira [9], who calculated that the power dissipation of a single *V. convallaria* was maintained at 1.2–1.6 nW over one order of magnitude change in the viscosity (Figure 10B inset). They also evaluated that the energy conversion efficiency of the spasmoneme was limited to 18%. However, it is likely impossible to estimate the isometric tension of the spasmoneme by increasing the medium viscosity because *Vorticella* can contract to its full extent even in highly viscous media. 

**Figure 11:**
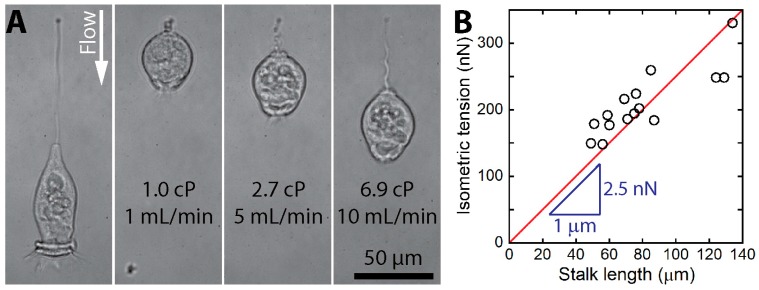
Isometric force measurements of live *V. convallaria* (reproduced from [16]). (**A**) *V. convallaria* stalled by viscous drag in a microchannel. The leftmost picture shows extended state, and the subsequent show contracted states under different flow conditions; (**B**) measured isometric tensions increased from 150 to 330 nN with a linear dependence on the stalk length, which suggests that the 1 μm of the spasmoneme can generate a tension of up to ~2.5 nN.

True measurements of the isometric tension are possible only when a stall or resistance force is directly exerted on contracting *Vorticella*. Recently, Ryu et al. employed a microfluidic channel to hydrodynamically stall the stalk contraction of *V. convallaria* [16]. Drag forces applied by controlled fluid flow opposing contraction were high enough to overcome the *Vorticella* contractility and stop it short of completion (Figure 11A). The estimated isometric tension ranged from 150 to 330 nN, and it appeared to increase in proportion to the stalk length (Figure 11B). This linear dependence of the isometric tension on the stalk length suggests that the 1 μm of the spasmoneme could generate a tension of up to ~2.5 nN.

Stall force has also been measured by suctioning the *Vorticella* cell body to a micropipette and calculating the spasmonemal force from pipette stiffness and deflection or pressure difference applied by the pipette. Both France [122] and Apolinar-Iribe et al. [123] employed a micropipette and measured the contractile force of live *Vorticella* to be in the hundreds of nanonewtons. 

### 3.4. Comparison with Other Motors

It is worthwhile to compare the contractility of the *Vorticella* spasmoneme with other contractile systems. In addition to protozoa with Ca^2+^-powered contractile organelles, leguminous plants have 2–4 μm diameter Ca^2+^-dependent contractile protein bodies in their sieve tubes, forisomes [26,124,125,126,127]. Forisomes repeat contraction-relaxation cycles responding to changes in Ca^2+^ concentration in ATP-depleted environments [128]. Isolated forisomes could develop forces of 50–120 nN [129,130,131,132], which is similar to the isometric tension of the spasmoneme. 

**Figure 12:**
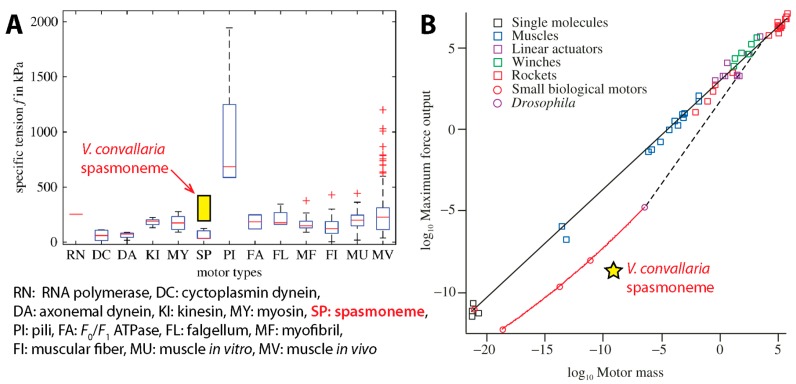
Comparison of the *Vorticella* spasmoneme with other motors. (**A**) Tensile stress (reproduced from [133]). Because the peak tension of live *V. convallaria* [14] and the isometric tension of extracted *V. convallaria* [23] are considered in [133], the isometric tensions of live *V. convallaria* [16] are included for rigorous comparison; (**B**) maximum force output (N) with respect to their mass (kg) (reproduced from [134,135]).

Considering that the spasmoneme was regarded as a primitive type of muscle, its maximum tension should be compared with that of skeletal muscle. Converted to tension per unit area (i.e., tensile stress) using the spasmoneme diameter of ~1 μm, Ryu et al.’s isometric tension measurement becomes 190–420 kPa [16], which is similar to the isometric tension of vertebrate striated muscles, 200–300 kPa [61,133]. When compared to other biological motors such as myosin, dynein and kinesin, the *Vorticella* spasmoneme is found to be similar to or outperform these motors in terms of tensile stress (Figure 12A). 

The above comparison considers differences in size or cross-sectional area among motors using tensile stress, and it is also meaningful to take into account the mass of motors. As shown by Ryu et al. [16], the *Vorticella* spasmoneme can generate the isometric tension of ~2.5 × 10^−9^ N per 1 μm length (Figure 11B). Because the average cellular density of *V. convallaria* was estimated to be 1.04 g/cm^3^ [122], the mass of the 1 μm-long spasmoneme is calculated to be 8.17 × 10^−10^ kg. When compared to other biological motors of similar mass, the *Vorticella* spasmoneme appears to underperform (Figure 12B) [134,135].

Conversely the *Vorticella* spasmoneme surpasses other motors in terms of specific power output (power per unit mass). Previously, Mahadevan and Matsudaira evaluated that the specific power of the spasmoneme is higher than that of typical passenger car engines (~0.3 W/g) [15]. For more rigorous comparison, we have calculated the specific power of the spasmoneme using the CFD-based result of Ryu and Matsudaira [9]: the maximum power dissipation of the ~117 μm-long *Vorticella* stalk was 1.56 nW. This value corresponds to 16.3 W/g when it is assumed that the spasmoneme length is close to the stalk length. This specific power value is four times the estimation of Mahadevan and Matsudaira, and is about two orders of magnitude higher than the specific power of car engines. This specific power of the *Vorticella* spasmoneme is exceptionally high even compared to mammalian skeletal muscle (0.28 W/g) and polymer-type engineered actuators (0.001–3.6 W/kg) [136,137]. 

### 3.5. Possible Engineering Applications

Because *Vorticella* stalks respond to concentration changes in Ca^2+^ or divalent cations in the medium, such stalks can operate as Ca^2+^-responsive actuators [20,106]. Similarly, pH-responsive polymers change their shape in response to the pH value of the medium, and thus their applications suggest possible engineering applications of *Vorticella* stalks in microscale devices [138,139], such as actuating microvalves [140,141] and microlenses [142,143]. 

**Figure 13:**
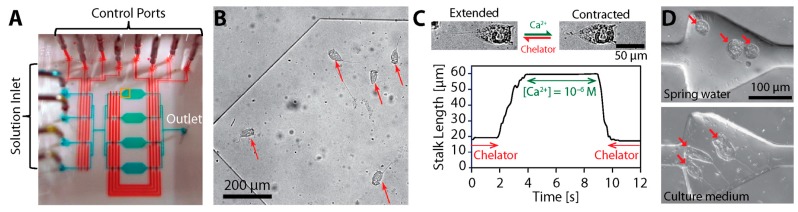
*V. convallaria* in a microfluidic system. (**A**) Microfluidic device for chemical control of extracted *V. convallaria*; (**B**) *V. convallaria* cells cultured in the device; (**C**) length change in a *Vorticella* stalk in a different [Ca^2+^]_free_ (A–C reproduced from [145]); (**D**) *V. convallaria* cells in a single chamber of which the size is comparable to the size of the cell (reproduced from [144]).

Nagai et al. demonstrated the responsive actuation of extracted *V. convallaria* cells in a microscale environment by modulating [Ca^2+^]_free_ using microfluidics (Figure 13) [144,145], and suggested a potential to construct Ca^2+^-responsive and Ca^2+^-powered microsystems based on *Vorticellae*. Then, Nagai et al. developed a Ca^2+^-responsive valve using *Vorticella*, for which a *Vorticella* cell was placed in a microchamber connected with a flow channel, and the extracted cell acted as a valve to open or block the channel depending on [Ca^2+^]_free_ [146]. 

Additionally, micro-objects were successfully attached to live *Vorticella* using biocompatible glues such as streptavidin-biotin binding [147] and poly-l-lysine coating [148], and their reciprocal motion was achieved from the stalk motion of *Vorticella*. Therefore, such binding methods prove that harnessing live *Vorticella* for microsystem application is feasible. In addition, the motion of live *Vorticella* can potentially be controlled by mechanical or electrical stimulation [11,105,119,149]. 

## 4. Discussion and Future Outlook

Microorganisms, such as bacteria or protozoa, have been integrated in microsystems to carry out specific functions, such as sensing and actuation, based on their cellular functionalities [29,150,151,152]. This is because such unicellular animals can self-replicate, perform multiple functions simultaneously, convert chemical energy into mechanical energy, functionally adapt to their environment, and be controlled through external stimuli. Because this approach requires sustaining the life of the integrated microorganisms, ones that can be easily cultured and maintained in various environmental conditions have been tested and utilized. Because *Vorticella* satisfies the above requirements, the ciliate is found to be a viable microorganism to be integrated in microsystems for flow control and actuation. 

Flagellated bacteria have been employed for flow control and pumping in microfluidic systems. It was estimated that *Escherichia coli* rotating at 10 rounds per second could generate a flow rate of 1.7 × 10^−5^ mm^3^/s [153], and tethered *Serratia marcescens* cells were observed to produce a flow rate of 3 × 10^−4^ mm^3^/s [100]. Considering that the oral cilia of a single *Vorticella* cell can transport water at a flow rate of 3 × 10^−4^ mm^3^/s [6], using multiple *Vorticella* cells would generate higher volume flow rates to control fluid flows. 

For actuation, larger microorganisms have been tested in addition to bacteria [154]. A biflagellate alga *Chlamydomonas reinhardtii* was shown to be able to carry microscale cargo at speeds of 100–200 μm/s [155] and to generate force of 10–37 pN [156]. The swimming of a larger ciliate protozoan *Paramecium caudatum* was maneuvered to be used as a microrobot, and it was observed to swim at the maximum speed of about 2 mm/s and to generate forces of about 27 nN [157]. Compared with these microorganisms, live *Vorticella* has better actuation capabilities because it can contract faster with higher forces (Table 1 and Table 2). Also, utilizing extracted *Vorticella* provides a unique actuation strategy to control its stalk contraction by adjusting external Ca^2+^ levels (Figure 13) and to convert chemical energy from Ca^2+^ binding to mechanical forces and motions. 

However, it needs to be noticed that stalked *Vorticella* may not be directly compared with freely moving *C. reinhardtii* and *P. caudatum*. In the free swimming telotroch form, a stalkless *Vorticella* (or telotroch) swims in water using aboral cilia around the scopula [4,158]. When *Vorticella* reproduces through budding, a daughter *Vorticella* leaves its mother zooid and becomes a telotroch. In other cases, a sessile stalked *Vorticella* turns into the telotroch form when it abandons its stalk presumably to change its habitat. Upon finding a suitable habitat, the *Vorticella* telotroch attaches, grows a stalk from the scopula, and transforms into the sessile stalked trophont form [159]. Although swimming *Vorticella* cells could be utilized similarly to *C. reinhardtii* and *P. caudatum*, the swimming of stalkless *Vorticella* has been rarely studied. If swimming *Vorticellae* are to be used, furthermore, their stalk formation will need to be inhibited [160].

More efficient uses of *Vorticella* cells in microsystems require controlling the animal’s motion. Because certain microorganisms move responding to an environmental stimulus, they can be controlled based on such taxis responses. For instance, the swimming of *Paramecium* was controlled using electric field based on its galvanotaxis [157,161,162], and *Chlamydomonas* was guided by light based on its phototaxis [155]. However, little is known about what types of taxis responses *Vorticella* shows. Instead, it seems more probable to use magnetic particles and magnetic field to control *Vorticella*, as applied for controlling a ciliate protozoan *Tetrahymena pyriformis* [163], because such particles can be attached to or engulfed by *Vorticella* [147,148]. Controlling the stalk contraction of *Vorticella* is also critical for utilizing the animal as a bioactuator. The contraction of live *Vorticella* stalks can be induced by mechanical or electrical stimulation [11,105,119,149], and extracted *Vorticella* stalks can be chemically controlled (Figure 8 and Figure 13) [17,20,21,23,145].

Using *Vorticellae* in microdevices has challenges that the viability of the live cells needs to be well maintained and that the extracted cells will denature gradually. Instead, the oral cilia and stalk of *Vorticella* can be mimicked to create similarly functioning devices or materials. Bioinspired cilia have been developed to create and control fluid flow in microsystems [85], and self-assembled cilia-like microtubule bundles are shown to beat and produce metachronal waves similar to cilia [97]. If such artificial cilia can be arranged and controlled to create circular metachronal waves mimicking the oral cilia band of *Vorticella* (Figure 3C), they would produce circulating flows similar to the feeding current of *Vorticella* (Figure 4). Similarly, if the biophysical mechanism of the spasmonemal contraction is unraveled, the Ca^2+^-responsive nanofibrils of the spasmoneme could be reconstructed in vitro to create spasmin-based nanoactuators, or the contraction mechanism could inspire inventing similar responsive polymers [27]. Also, the structure of the *Vorticella* stalk, which transforms the linear contraction of the spasmoneme into the helical coiling of the stalk (Figure 7A), can be employed for an effective design of bioinspired soft robots [164,165,166].

## 5. Conclusions

Using microorganisms either directly in microscale systems or as the inspiration to engineer similar biomimetic systems has the possibility to open up new frontiers in micromachinery. This is because these organisms have the unique ability to sense and actuate simultaneously. This review paper specifically introduces the sessile ciliate protozoan *Vorticella* as a novel and promising model organism for bio-inspired engineering, microscale fluid controllers, and calcium-sensitive and calcium-powered actuators.

*Vorticella* consists of a zooid with oral cilia that generate a feeding current and a slender stalk that anchors to a habitat surface. The oral cilia of *Vorticella* beat sequentially to form metachronal waves and generate circulating water flow with a speed of 0.4–2.5 mm/s, which translates food particles toward the zooid. This flow-generating capability of the *Vorticella* cilia has been successfully tested as the micromixer in microfluidic systems, but the effect of surrounding boundaries on the generated circulating flow should be considered. 

The stalk of *Vorticella* coils in a few milliseconds, translating the zooid at a speed of 15–90 mm/s, which is driven by its calcium-powered contractile organelle, the spasmoneme. Because of its unique capability and energy source, the spasmoneme is regarded as a model organelle for bioinspired actuators. The spasmoneme can generate contractile forces of 30–330 nN powered by the binding of calcium ions. This spasmonemal force is comparable to the maximal force of other motors, but it has specific power much higher than other biological and man-made motors. The calcium-sensitive contractility of *Vorticella* has been tested for moving microscale objects in microfluidic systems. Identifying the biophysical mechanism of the spasmonemal contraction, as well as determining methods for controlled culture of *Vorticella* at desired locations in microfluidic devices, will increase the great potential of *Vorticella* within the realm of bioinspired actuators. 

## Figures and Tables

**Table 1:** Key dynamics parameters of the stalk contraction of live *Vorticella* and its relatives (in water).

Reference	Cell	Stalk Length	Peak Contraction Speed	Time for Peak Speed	Contraction Time	Latent Period	Contraction Propagation Speed
Ueda (1954) [32]	*C. polypinum*	1.05–1.40 mm	17.8–24.7 cm/s	-	7–10 ms	2–3 ms	-
Sugi (1960) [33]	*C. polypinum*	0.8–2.2 mm	-	-	-	1–4 ms	20–50 cm/s
Jones et al. (1970) [119]	*V. difficilis*	140–200 μm	1.4–5.6 cm/s	-	~10 ms	-	3.5–10.5 cm/s
Weis-Fogh and Amos (1972) [38]	*Z. geniculatum*	1 mm	110 length/s (11 cm/s)	-	5 ms	-	-
Rahat et al. (1973) [39]	*Carchesium*	-	-	-	40–60 ms	-	-
Katoh and Naitoh (1992) [11]	*Vorticella*	-	6.7 cm/s	-	-	1.7 ms	-
Moriyama et al. (1998) [12]	*V. convallaria*	~250 μm	8.8 cm/s	2 ms	~9 ms	-	-
Upadhyaya et al. (2008) [14]	*V. convallaria*	150 μm	~6 cm/s	~2.5 ms	~6 ms	-	~10 cm/s
Ryu and Matsudaira (2010) [9]	*V. convallaria*	117 μm	5.8 cm/s	1 ms	3.9 ms	-	-
Kamiguri et al. (2012) [120]	*Vorticella*	-	4.3–7.5 cm/s	-	-	-	-

**Table 2:** Tension and energetics of the stalk contraction of *Vorticella* and its relatives (in water).

Reference	Cell	Stalk (st) or Spasmoneme (sp) Dimension	Contractile Force or Tension	Isometric Force or Tension	Work	Instantaneous or Maximum Power Output	Energy Efficiency
Ueda (1952) [31]	*C. polypinum* (Live)	1–2.7 mm long 12–15 μm diameter (st)	0.092–0.132 mg (0.9–1.3 μN) 65–88 g/cm^2^ (6.3–8.6 kPa)	-	-	-	-
Hoffman-Berling (1958) [17]	*Vorticella* (Live)	-	~10 kPa [42]	-	-	~2.8 W/g [42]	-
Amos (1971) [13]	*Vorticella* (Live)	80 μm long 1 μm diameter (sp) [44]	8.7 Nn (11 kPa [44]) 20–30 g/cm^2^ [39] (1.96–2.94 kPa)	-	0.69 pJ	2300 cal/g/h [39] (2.67 W/g)	-
Weis-Fogh and Amos (1972) [38]	*Z. geniculatum* (Glycerinated)	1 mm long 30 μm diameter (sp)	30 kPa [42]	-	-	~4 W/g [42]	-
Rahat et al. (1973) [39]	*Carchesium* (Live)	5 μm diameter (sp)	400–800 g/cm^2^ (39.2–78.4 kPa)	-	-	-	-
Moriyama et al. (1996) [23]	*V. convallaria* (Glycerinated)	1.0–1.2 μm diameter (sp)	-	40 nN (avg) 120 nN (max) (35–51 kPa)	-	-	~7%
Moriyama et al. (1998) [12]	*V. convallaria* (Live)	~250 μm long	55.8 nN	-	-	-	-
France (2007) [122]	*V. convallaria* (Live)	-	109–142 nN	-	1.93–2.59 pJ	0.70–0.93 nW	-
Upadhyaya et al. (2008) [14]	*V. convallaria* (Live)	150 μm long	~30 nN	-	-	1–3 nW	-
Ryu and Matsudaira (2010) [9]	*V. convallaria* (Live)	117 μm long	28 nN	-	1.64 pJ	1.6 nW	8%
Apolinar-Iribe et al. (2010) [123]	*Vorticella* (Live)	-	117.7 nN	-	-	-	-
Ryu et al. (2012) [16]	*V. convallaria* (Live)	49–134 μm long	-	150–330 nN	-	-	-

## Abbreviations

The following abbreviations are used in this manuscript:

ATP	Adenosine TriPhosphate
CFD	Computational Fluid Dynamics
ICL	InfraCilliary Lattice
PIV	Particle Image Velocimetry
PTV	Particle Tracking Velocimetry
TEM	Transmission Electron Microscope
